# Mucocutaneous diseases with manifestations in the head and neck region: 24 years of experience in a Dermatology service

**DOI:** 10.4317/medoral.25549

**Published:** 2023-01-15

**Authors:** Weslay Rodrigues da Silva, Reydson Alcides de de Lima-Souza, Leorik Pereira da Silva, Luiz Gonzaga de Castro e Souza Filho, Luciano Tavares Montenegro, Déborah Pitta Paraíso Iglesias

**Affiliations:** 1 Department of Intensive Care Unit, Real Hospital Português (RHP), Recife, PE, Brazil; 2 Department of Oral Diagnosis, State University of Campinas (UNICAMP), Piracicaba, SP, Brazil; 3 Academic Unit of Biological Sciences, Federal University of Campina Grnade (UFCG), Patos, PB, Brazil; 4 Department of Tropical Medicine, Federal University of Pernambuco (UFPE), Recife, PE, Brazil; 5 Department of Pathology, Federal University of Pernambuco (UFPE), Recife, PE, Brazil

## Abstract

**Background:**

This study aimed to evaluate the clinicopathological features of mucocutaneous diseases with manifestation in the head and neck region.

**Material and Methods:**

A retrospective analysis of a dermatology reference center database was carried out. Over 24 years. Clinicopathological data were collected from medical records and the data was analyzed by descriptive statistics.

**Results:**

A total of 11.538 medical records were analyzed, being 152 cases of mucocutaneous diseases with manifestations in the head and neck region. Cutaneous lupus erythematosus was the most prevalent diagnosis (66.4%). Face (44.1%), females (79.6%), and patients with 45 years mean age were the most common features. In the oral cavity, the most affected region was the buccal mucosa (37.5%).

**Conclusions:**

Mucocutaneous diseases with head and neck manifestation were rare in the sample analyzed (1.3%), with cutaneous lupus erythematosus and lichen planus being the most common lesions in this region.

** Key words:**Mucocutaneous diseases, head and neck, oral lesion, lichen planus, lupus erythematosus, pemphigus, pemphigoid.

## Introduction

Dermatological diseases are represented by primary diseases of the skin and cutaneous manifestations of systemic diseases (1). Mucocutaneous involvement in dermatological diseases may be an immunological or inflammatory etiology. They are noted in several situations and, when compiling these conditions, it is possible to notice variations in the etiopathogenesis and clinical course (2).

Although there are many mucocutaneous diseases, such as pemphigus, pemphigoid, acquired bullous epidermolysis, lichen planus, erythema multiforme, and lupus erythematosus, when it’s affected the head and neck region, it is common for studies to describe epidemiological data for only one disease (3-5).

The knowledge and characterization of mucocutaneous diseases are essential to the diagnosis and development of therapeutic strategies. Some diseases of this group are significantly associated with morbidity and mortality (1,2). This is an important aspect, especially in those with head and neck manifestations, as they may bring several functional and aesthetic comorbidities to the patients (6,7). Thus, this study aimed to analyze the clinicopathological data of mucocutaneous diseases with head and neck manifestations diagnosed in a dermatological reference service in 24 years.

## Material and Methods

A retrospective study of 24 years (1994-2018) was conducted in the Dermatology Ambulatory of the Clinics Hospital, at the Federal University of Pernambuco, a public university in Brazil. This study was approved by the Institutional Review Board (Protocol number: 3.321.187) and complied with the Declaration of Helsinki.

Cases of mucocutaneous diseases with head and neck manifestations of female and male patients diagnosed at a dermatology service were used. We collected from medical records the following clinicopathological data: Patient’s sex, age, anatomical site, clinical impression, and histologic diagnosis. The inclusion criteria considered all cases of mucocutaneous diseases with head and neck manifestations. Records without histopathological diagnosis and/or indeterminate diagnosis and insufficient data were excluded from the study.

The collected data were organized in Excel (Microsoft Office) spreadsheets and tabulated in SPSS (Statistical Package for Social Sciences), version 22. Data was analyzed by descriptive statistics, with the relative and absolute distributions of the variables.

## Results

Over 24 years period, 11.538 cases of dermatological lesions were diagnosed in the dermatology reference center. Of 498 (4.3%) cases of immunological /inflammatory diseases with mucocutaneous involvement, 152 (1.3%) cases occurred in the head and neck region. All 152 cases were following the eligibility criteria of this study. The mucocutaneous diseases with head and neck manifestations comprise cutaneous lupus erythematosus [66.5%], lichen planus [19], pemphigus [12.5], and pemphigoid lesions [2%]. All clinical impressions were in accordance with histopathological diagnosis (Table 1).

Females were more affected 121 (79.6%) when compared to males 31 (20.4%), with a 3.9:1 female:male ratio. The age of patients ranged from 24 to 83 years, with an average age of 45 (SD ± 14.5), with the fourth and fifth decades of life the most affected. Regarding the anatomical site, the face was the most common location, corresponding to 44.1% of the cases, followed by the scalp (25.1%) and oral cavity (23%). The face and oral cavity were divided into sites, with the nose (35.8%) and buccal mucosa (37.1%) being the most affected site respectively (Table 2).

**Table 1:**
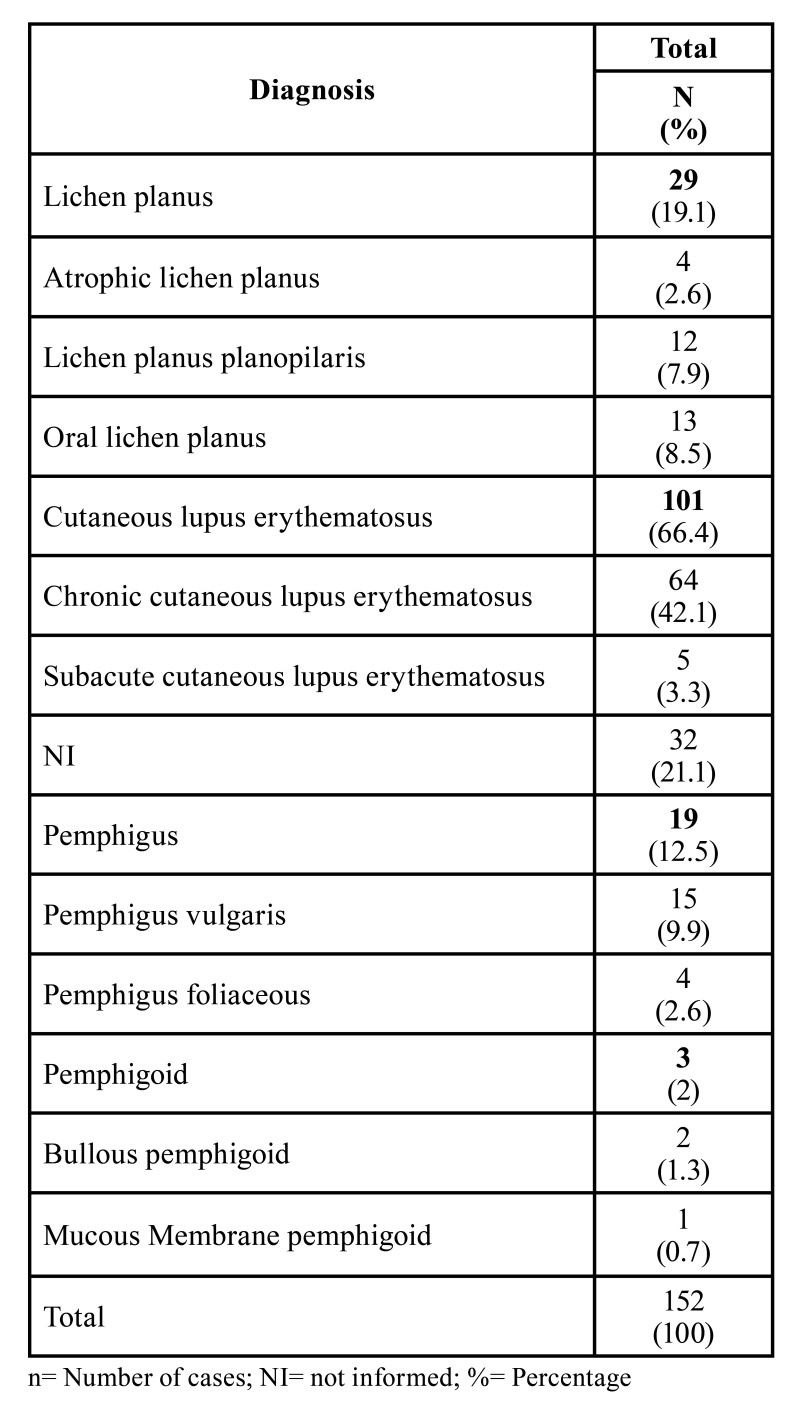
Classification of lesions according to histological diagnosis/clinical type.

**Table 2:**
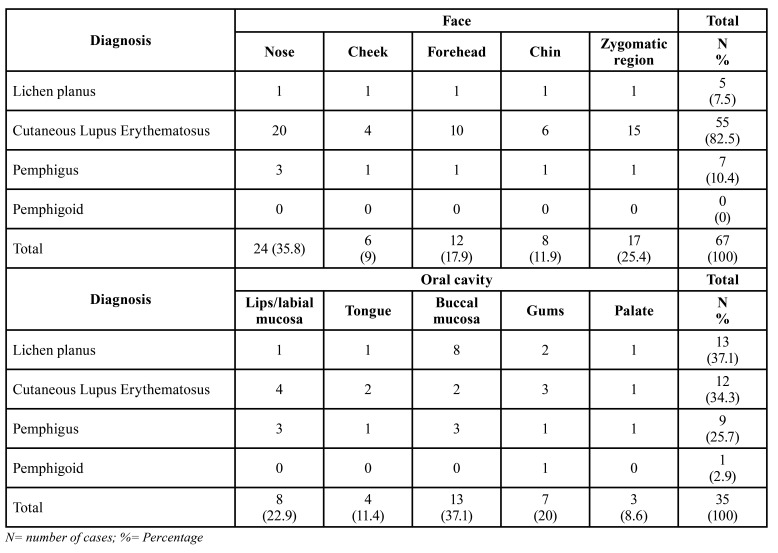
Distribution of lesions of the face and the oral cavity.

## Discussion

Concerning head and neck diseases, mucocutaneous diseases with manifestation at this site represent an important group of lesions with a varied clinical course. Some lesions may present overlapping features, leading to diagnostic difficulties. Thus, clinical and epidemiological studies are essential to assist in the diagnosis at the early stages of these diseases.

In the present study, cutaneous lupus erythematosus was the most common condition, representing about 65% of the cases with head and neck manifestations. The cutaneous form is one of the most varied presentations of lupus erythematosus, an autoimmune mucocutaneous disease (1,8). The skin is the organ most affected by the cutaneous presentation and may represent the only manifestation site. This form can be classified as acute, subacute, and chronic (9). Our data reveals similarities with what has been described in the literature: a predilection for middle-aged women and manifestations in the face (1,8).

When occurring on the face, habitually it is present as a butterfly shape, erythematous rash over the cheeks, and nasal bridge, strongly supporting the lupus erythematosus diagnosis (9). Oral lesions may appear in more than 40% of patients with lupus erythematosus and some cases may be the only lesions seen in these patients (10). In the oral cavity, however, the clinical presentation is nonspecific, present as an ulcer or eroded region with white streaks on the border mainly involving the palate, tongue, labial, and buccal mucosa (2,10-12). In our sample, oral represented 12% of the cutaneous lupus erythematosus with a predilection for the lip/labial mucosa and gums.

Lichen planus is a dermatosis whose pathogenesis is not completely known but is believed to be immunologically mediated (1,13). It affects patients of all ages and sex but presents a predilection in middle-aged female patients (13). The lesions can assume a localized or generalized form, and occur mainly in the flexor surfaces of the extremities, dorsal aspect of the trunk, and scalp (1,11,14). Our data, showed a higher predilection for females, with a female:male ratio of 8.7:1. Besides the mean age following the literature, elderly people were more affected. These results lead us to suggest that epidemiological data on lichen planus may vary according to the population studied.

In addition to the classic variant of lichen planus, several variants are described based on both morphological and topographic distribution in oral, nail, linear, annular, atrophic, hypertrophic, inverse, eruptive, bullous, ulcerative, pigmentosus, planopilaris, vulvovaginal, actinic, lichen planus-lupus erythematosus overlap syndrome, and lichen planus pemphigoids (1,11,14).

Although up to 60% of patients with lichen planus may have an oral lesion, only 15% of patients with oral lichen planus develop skin lesions. In these cases, the skin lesions develop after a few months of the appearance of the oral lesions (1,15). Unlikely classical lichen planus, the oral lichen planus usually presents as symmetric lacy distribution of whitish-gray lines (Wickham's striae), sometimes with small white papules or plaques disposed on an erythematous background, mainly in the buccal mucosa (6,16-18). The clinical appearance and high incidence of this variant (1,6,11,14,16-18) may be contributed to the identification of oral lichen planus in our sample, being the most form of lichen planus (44.8%). In addition, these patients with oral lichen planus need long-term monitoring because of the relative risk of malignant transformation, especially associated with the erosive subtype (1,19).

Pemphigus is a dermatological disease with mucocutaneous involvement characterized by the formation of autoantibodies against desmosomal components known as desmoglein 1 and desmoglein 3 (1,20,21). Usually, this lesion affects the trunk, scalp, and face of middle-aged females (4,6,22,23). While several different variants of pemphigus have been described, pemphigus vulgaris stands out as the most prevalent variant, comprising up to 70% of all cases of pemphigus (20,21). In our casuistic, the results are following the literature with pemphigus vulgaris comprising about 80% of all the pemphigus cases. Furthermore, was evidenced a predilection for females when compared with a female:male ratio of 2.8:1.

The oral cavity is the most frequent mucosal involved, not only in the head and neck region but in all body parts (1,7,21,23). Our sample demonstrated similar results, with the oral cavity being the most commonly affected site, mainly in buccal mucosa and lips/labial mucosa. Notoriously, the oral cavity manifestation may represent the first sign of the disease and in 50% of cases this region is the only affected site, being the buccal mucosa, palate, and labial mucosa/lips more frequently related (1,5,6,21,23,24).

Pemphigoid corresponds to a group of dermatological diseases, characterized by the formation of autoantibodies against hemidesmosomes (25-27). This group may be divided into bullous and mucous membrane pemphigoid (26) however, besides these lesions sharing some characteristics, they present significant differences in clinical course, treatment, and prognosis (1,25-28). In general, they preferentially affect women between the sixth and seventh decades of life (25,27). In the mucous membrane pemphigoid, the gingiva is affected in 94% of cases, causing desquamative gingivitis (24). In our casuistic, only three patients were diagnosed with pemphigoid diseases, and the oral pemphigoid case presented as desquamative gingivitis, a clinical term given to the gingival manifestation of mucocutaneous diseases (5,24,29).

Few studies in the literature report the association of clinical diagnoses with histological diagnoses. In our study, all clinical diagnoses agreed with the histological diagnoses. Despite this, we emphasize the importance of performing histopathological examinations to confirm the diagnosis and avoid inappropriate and ineffective conduct.

The present study has several strengths that may serve as a basis for future studies that aim to evaluate the clinicopathological features of mucocutaneous diseases with head and neck manifestations. Aside from that, some limitations need to be considered. First, the Stomatology service at the university may be the reason for a few diagnoses’ types of mucocutaneous lesions in the head and neck region. Second, for the sake of this, the real profile of the head and neck mucocutaneous lesion was affected. Lastly, because this was a retrospective study of a dermatology service, accurate and detailed information, especially regarding oral lesions, such as clinical appearance, time of evolution, and symptomatology, among others, was not available. We recognize and emphasize the importance of Dermatology services reporting complete information about oral lesions, since such lesions may be the first sign of the disease.

## Conclusions

In conclusion, the results of this study mucocutaneous diseases with head and neck manifestation were rare in the sample analyzed (1.3%), with cutaneous lupus erythematosus and lichen planus being the most common lesions in this region. Thus, studies that access the clinicopathological and epidemiological data of mucocutaneous diseases with head and neck manifestations are encouraged, once this region may represent the first sign of disease. Along with this, the knowledge of clinicopathological features assists in the diagnosis and development of therapeutic strategies more conservative and effective.
